# Novel sources of resistance to Septoria nodorum blotch in the Vavilov wheat collection identified by genome-wide association studies

**DOI:** 10.1007/s00122-018-3073-y

**Published:** 2018-02-22

**Authors:** Huyen T. T. Phan, Kasia Rybak, Stefania Bertazzoni, Eiko Furuki, Eric Dinglasan, Lee T. Hickey, Richard P. Oliver, Kar-Chun Tan

**Affiliations:** 1 Centre for Crop and Disease Management, School of Molecular and Life Sciences, Curtin University, Bentley, WA Australia; 2 Queensland Alliance for Agriculture and Food Innovation, The University of Queensland, St. Lucia, QLD Australia

## Abstract

**Key message:**

The fungus *Parastagonospora nodorum* causes Septoria nodorum blotch (SNB) of wheat. A genetically diverse wheat panel was used to dissect the complexity of SNB and identify novel sources of resistance.

**Abstract:**

The fungus *Parastagonospora nodorum* is the causal agent of Septoria nodorum blotch (SNB) of wheat. The pathosystem is mediated by multiple fungal necrotrophic effector–host sensitivity gene interactions that include SnToxA–*Tsn1*, SnTox1–*Snn1,* and SnTox3–*Snn3*. A *P. nodorum* strain lacking *SnToxA*, *SnTox1,* and *SnTox3* (*toxa13*) retained wild-type-like ability to infect some modern wheat cultivars, suggesting evidence of other effector-mediated susceptibility gene interactions or the lack of host resistance genes. To identify genomic regions harbouring such loci, we examined a panel of 295 historic wheat accessions from the N. I. Vavilov Institute of Plant Genetic Resources in Russia, which is comprised of genetically diverse landraces and breeding lines registered from 1920 to 1990. The wheat panel was subjected to effector bioassays, infection with *P. nodorum* wild type (SN15) and *toxa13*. In general, SN15 was more virulent than *toxa13.* Insensitivity to all three effectors contributed significantly to resistance against SN15, but not *toxa13*. Genome-wide association studies using phenotypes from SN15 infection detected quantitative trait loci (QTL) on chromosomes 1BS (*Snn1*), 2DS, 5AS, 5BS (*Snn3*), 3AL, 4AL, 4BS, and 7AS. For *toxa13* infection, a QTL was detected on 5AS (similar to SN15), plus two additional QTL on 2DL and 7DL. Analysis of resistance phenotypes indicated that plant breeders may have inadvertently selected for effector insensitivity from 1940 onwards. We identify accessions that can be used to develop bi-parental mapping populations to characterise resistance-associated alleles for subsequent introgression into modern bread wheat to minimise the impact of SNB.

**Electronic supplementary material:**

The online version of this article (10.1007/s00122-018-3073-y) contains supplementary material, which is available to authorized users.

## Introduction

The fungus *Parastagonospora* (syn. *Stagonospora*; *Phaeosphaeria*, *Septoria*) *nodorum* (Berk.) Quaedvlieg, Verkley & Crous is the causal agent of Septoria nodorum blotch (SNB) of wheat (*Triticum aestivum*) (Quaedvlieg et al. 2013; Solomon et al. 2006). The pathogen causes foliar and glume damage that results in significant yield losses in many wheat growing areas around the world (Eyal et al. 1987; Oliver et al. 2009). In Australia, SNB is responsible for losses up to AUD$108 million (~ USD$86.7 million) per year to the cereal industry (Murray and Brennan 2009). SNB is largely governed by a series of proteinaceous fungal necrotrophic effectors (NEs) that cause necrosis and/or chlorosis on wheat lines possessing matching dominant susceptibility genes through effector-triggered susceptibility (Tan et al. 2010). Thus far, three single copy genes that encode NEs have been identified in *P. nodorum*. *SnToxA* encodes a 13.2 kDa mature protein. Sensitivity to SnToxA is conferred by the expression of *Tsn1* which is localised in the long arm of chromosome 5B (Friesen et al. 2006). *Tsn1* encodes a nucleotide binding site leucine-rich repeat protein with a protein kinase domain (Faris et al. 2010). *SnTox1* encodes a 10.3 kDa small cysteine-rich protein with a putative chitin-binding domain. The effector is indirectly recognised by a receptor kinase encoded by *Snn1* which is located on the short arm of chromosome 1B (Liu et al. 2012). Recognition results in cell death and disease. SnTox1 has chitin-binding properties and protects the fungus against wheat chitinases (Liu et al. 2016). *SnTox3* encodes a 17.5 kDa mature protein that contains six cysteines (Liu et al. 2009). SnTox3 confers necrosis and subsequent effector-triggered susceptibility on wheat containing *Snn3* located on the short arm of chromosome 5B and its homolog in *Aegilops tauschii* is on chromosome 5DS (Zhang et al. 2011). Unlike *Tsn1* and *Snn1*, *Snn3* is yet to be cloned and characterised. Gene expression analysis indicates that *SnToxA*, *SnTox1,* and *SnTox3* are highly expressed during early infection coinciding with host penetration (Rybak et al. 2017). Furthermore, biochemical and genetic analyses indicate *P. nodorum* possesses several undiscovered NEs that may also function as inverse gene-for-gene determinants in SNB (Friesen et al. 2008a).

SNB is a complex disease. It is likely that an interplay of NE–host dominant susceptibility receptor interactions and host resistance loci quantitatively contribute to SNB (Aguilar et al. 2005; Friesen and Faris 2010; Shankar et al. 2008). We previously set out to examine the collective role of SnToxA–*Tsn1*, SnTox1–*Snn1,* and SnTox3–*Snn3* interactions in SNB of Australian wheat through the creation of a strain of *P. nodorum* called *toxa13*, derived from wild-type SN15, where *SnToxA*, *SnTox1,* and *SnTox3* were deleted using homologous recombination (Tan et al. 2015). To our surprise, removal of these effector genes did not reduce virulence on several widely-grown Australian bread wheat cultivars at the seedling stage. Furthermore, *toxa13* secreted a chlorosis inducing factor(s) that appeared to compensate for the loss of *SnToxA*, *SnTox1,* and *SnTox3*. Therefore, these wheat lines either lacked a basal resistance mechanism against SNB or an undiscovered effector–dominant susceptibility gene interaction functioned to compensate for the loss of the three major effector genes (Tan et al. 2015).

To find novel sources of genetic resistance, we turned our attention to the genetic diversity found in wheat accessions that were developed over time for local adaptation before modern breeding. One of the earliest seed banks was established by Nikolai Ivanovich Vavilov (1887–1943), a prominent Soviet plant breeder and botanist. The N. I. Vavilov Institute of Plant Genetic Resources (VIR) located in Russia houses a vast collection of cereals, potatoes, and various other agricultural crops. As of today, 38,430 viable wheat accessions are preserved at VIR (Mitrofanova 2012). Of these, 29,290 are bread wheat, 6199 are durum, and 3022 are wild primitive wheat. Recently, Riaz et al. (2017c) assembled a panel of 295 bread wheat Vavilov accessions comprising of landraces, breeding lines, and cultivars collected from 28 countries between 1922 and 1990. This panel was genotyped using the Diversity Arrays Technology genotyping-by-sequencing (DArT-Seq) platform which revealed a high degree of allelic diversity compared to modern cultivars and elite lines from Australia to the International Maize and Wheat Improvement Centre (CIMMYT) in Mexico (Riaz et al. 2017c). Novel sources of adult plant resistance to the leaf rust fungus *Puccinia triticina* were identified through characterisation of this Vavilov diversity panel (Riaz et al. 2017a).

Most reports on the identification of genetic resistance to SNB carried out in the last 20 years were performed using bi-parental mapping populations (e.g., Aguilar et al. 2005; Arseniuk et al. 2004; Uphaus et al. 2007). Whilst this approach has yielded valuable information for a number of key QTL that can be pyramided for resistance, allelic variation in bi-parental mapping populations is limited to the parental lines used to generate the cross. Genome-wide association studies (GWAS) can overcome this limitation. For example, Gurung et al. (2014) used a panel of 528 diverse spring wheat landraces genotyped with single nucleotide polymorphism (SNP) markers for GWA to examine several major biotic diseases including SNB. QTL were identified on 2D, 3A, and 5B conferring seedling resistance against a *P. nodorum* isolate that lacked SnTox3 (Adhikari et al. 2011; Gurung et al. 2014). The 5B QTL corresponded to the well characterised *Tsn1* (Gurung et al. 2014; Liu et al. 2009) confirming the ability of GWAS to detect SNB QTL.

The aim of this study is to use a combination of wheat diversity, *P. nodorum* strains differing in effector gene profile and GWAS to further dissect the complexity of SNB in the presence and absence of SnToxA–*Tsn1*, SnTox1–*Snn1,* and SnTox3–*Snn3* interactions. Furthermore, the availability of collection data on many registered accessions enable us to make limited but insightful inferences on the spatial and temporal distribution of effector insensitivity in the Vavilov wheat collection.

## Materials and methods

### Biological material

The Vavilov wheat collection was subjected to *P. nodorum* infection and effector infiltration. Of the 295 available wheat accessions, 253 were evaluated for response to SNB and 259 were evaluated for effector sensitivity. The remaining accessions were omitted from analysis due to poor growth or lack of seeds during different stages of the experiment. In addition, 224 CIMMYT and International Centre for Agricultural Research in the Dry Areas (ICARDA), 47 Australian wheat cultivars and Chinese Spring were obtained from the Australian Grains Genebank (Horsham, Australia). These were subjected to *P. nodorum* infection or effector infiltration. All wheat lines were grown in 12 cm pots containing perlite and vermiculite (The Perlite and Vermiculite Factory, Australia) at 21 °C under a 12 h light and dark cycle for 2 weeks prior to manipulation.

All *P. nodorum* isolates were maintained on V8-PDA agar at 21 °C under a 12 h light and dark cycle for 2 weeks prior to manipulation (Phan et al. 2016).

### Effector expression and infiltration

Effector assays were performed using a simple leaf infiltration technique (Oliver et al. 2009). SnTox1 (SNOG_20078) and SnTox3 (SNOG_08981) effectors were produced in *Pichia pastoris* using the pGAPzA expression system (Thermo Fisher Scientific, MA, USA) (Liu et al. 2009, 2012). SnToxA was produced in *E. coli* using the pET system (Novagen) by the UQ Protein Expression Facility at the University of Queensland, Australia (Tan et al. 2012). All proteins were in 20 mM sodium phosphate buffer pH 7.0 prior to infiltration. A needleless one cc plastic syringe was used to infiltrate the expressed proteins into the first leaf of 2-week-old wheat seedlings. Plants were returned to the Conviron growth chamber for 4 days for SnToxA- and SnTox3-induced necrosis and 7 days for SnTox1. Wheat plants were visually evaluated for effector sensitivity using a scale of ‘0’ to ‘4’, where a score of 0 indicates no observable reactions; 1, mild chlorosis; 2, chlorosis; 3, chlorosis with mild necrosis; 4, necrosis (Tan et al. 2012). All infiltrations were carried out in biological triplicates where possible. SnToxA infiltration data were adapted from Dinglasan et al. (2018).

### Whole-plant infection assay

Seedling infections were performed using the whole-plant spray assay technique, described in Phan et al. (2016). Briefly, pycnidiospore inoculum was prepared to a concentration of 1 × 10^6^ spores/ml in 0.5% (w/v) gelatine. Wheat cultivars were planted in a completely randomised design in three replicates. Four-to-six seeds of each line were planted in a 120-cm-dimension pot and considered as a repeat. Two-week-old wheat seedlings were sprayed with the inoculum preparation using a hand-held air brush sprayer until runoff. Plants were placed in 100% relative humidity at 21 °C in the light for 72 h, followed by 7 days at 21 °C under a 12 h photoperiod prior to scoring. A score of ‘1’ indicates that no disease symptoms were observed and a score of ‘9’ indicates a fully necrotised plant. Infections were carried out in biological triplicates.

### Curation of the genotyping data

The raw data set comprised of 29,904 DArT and 18,827 SNP markers was originally obtained from DArT-seq Technology (Diversity Arrays Technology, Australia) by Riaz et al. (2017c). Markers with genetic location information available were selected for further filtering. However, only DArT markers were used for data analysis in Riaz et al. (2017c). In this study, further filtering was applied to both types of markers to remove markers with more than 10% missing values. Duplications between DArT and SNP clones were also excluded. When multiple SNPs were located on the same clone, only one was chosen based on least missing values, all other SNPs were omitted. Additional filtering steps discarded markers with minor allele frequencies ≤ 0.05 or ≥ 0.95 to avoid bias in association analysis due to imbalanced allele frequencies. The low proportion of missing marker data in the cleaned dataset was imputed using the missForest v1.4 package in R for missing data points (Stekhoven and Buehlmann 2016). Finally, co-segregating markers were removed to produce a final data set for GWAS comprising 10,176 DArT and 2710 SNP markers. A smaller subset of 6294 DArT and 1697 SNP markers, including only markers with less than 0.8 correlation coefficient with all other markers, was used for kinship analysis and population structure adjustment.

### Genome-wide association studies

Phenotypic data of the five qualitative traits examined in the Vavilov wheat accessions were used to identify marker–trait associations using Mixed Linear Model (MLMs) analyses accounting for the population structure and kinship as fixed effects using the package GWASpoly in R (Rosyara et al. 2016). To identify SNP/DArT markers with significant associations, two thresholds were applied for different phenotyping methods. For the effector-sensitivity assay where a single inverse gene-for-gene interaction was expected, the Bonferroni corrected *P* = 0.05 method [− log_10_(*P*) = 5.49] was used as described by Cockram et al. (2015). For whole-plant disease infection where more complex interactions contributed to the SNB severity among the Vavilov wheat accessions, an arbitrary threshold of − log_10_(*P*) = 3.5 was applied, similar to the approach adopted in recent GWAS studies by Qian et al. (2016) and Riaz et al. (2017b).

QTL locations were classified according to the consensus genetic map provided by Diversity Arrays Technology Pty Ltd (version 4.0) with the original data set. To further position these QTL on the wheat chromosome arms, marker sequences were used to search against the wheat genome assembly in EnsemblPlant (http://plants.ensembl.org/index.html; Clavijo et al. 2017) using blastn with the cut-off value of e^−18^. Manhattan plots were constructed to display the whole genome marker–trait associations using R ‘qqman’ package (Stephen Turner, https://cran.r-project.org/web/packages/qqman/qqman.pdf).

### Allele stacking analysis and haplotype construction

The effect of an accumulation of alleles that are associated with SNB resistance was determined by assigning accessions from the Vavilov wheat collection to groups based on the number of resistance-associated alleles. Since the reference allele used in GWASpoly package was “0”, we designated the resistance-associated allele for each significant marker based on the predicted effect of each QTL on the disease severity obtained from GWAS (Table 1). This assignment was then used to study the effect of QTL stacking on the severity of SNB caused by SN15 and *toxa13*. Vavilov accessions were grouped according to the number of resistance-associated alleles they carry.

**Table 1:** Summary of SNB and effector-sensitivity QTL identified from this study

Treatment	Marker^a^	Chromosome	Gene	Position (cM)^b^	*P* value	Allele for resistance	Score	Effect
SnToxA	1168841	5BL	*Tsn1*	137.096	3.548e−10	0	9.45	1.92
SnTox1	1268227	1BS	*Snn1*	7.025	9.120e−11	0	10.04	1.14
	1195188	1BS	*Snn1*	7.025	8.913e−08	0	7.05	1.05
	1099649	1BS	*Snn1*	7.025	1.097e−10	0	9.96	1.16
SnTox3	1126921	5BS	*Snn3*	4.119	7.586e−15	0	14.12	2.06
	1673475	5BS	*Snn3*	5.103	2.818e−13	0	12.55	1.87
	1151694	5BS	*Snn3*	5.452	9.550e−13	0	12.02	1.89
	1232949	5BS	*Snn3*	5.452	1e−15	0	15.00	2.07
	2276671	5BS	*Snn3*	6.153	4.074e−13	0	12.39	1.89
SN15 SNB	1268227	1BS	*Snn1*	7.025	5.623e−07	0	6.25	0.99
	1195188	1BS	*Snn1*	7.025	6.457e−05	0	4.19	0.87
	2248448	2DS	*Snn2* ^c^	195.480	2.951e−04	0	3.51	1.26
	1254900	3AL		209.700	2.512e−04	1	3.60	− 1.74
	1676515	4AL^d^		240.629	2.951e−04	1	3.53	− 1.21
	1104887	4BS		5.495	1.778e−04	1	3.75	− 0.98
	3939782	5AS		40.050	8.318e−05	0	4.08	1.03
	1126921	5BS	*Snn3*	4.119	6.310e−05	0	4.20	0.93
	1151694	5BS	*Snn3*	5.452	4.898e−05	0	4.31	0.93
	1232949	5BS	*Snn3*	5.452	2.239e−05	0	4.65	0.94
	2276671	5BS	*Snn3*	6.153	1.047e−04	0	3.98	0.87
	2374474	7AS		110.881	1.445e−04	1	3.84	− 1.12
*toxa13* SNB	1231149	2DL	*Snn7* ^c^	140.670	1.950e−04	0	4.01	0.75
	1126530	5AS		72.798	5.623e−05	0	4.25	0.87
	1118447	7DL		231.408	4.898e−05	0	4.27	0.85

Details of significantly associated markers, chromosomal location, − log_10_(*P*) score and effect for each marker are provided. Manhattan plots are presented in Supplemental data 3

^a^All markers shown are DArT

^b^As in the consensus map generated by Diversity Arrays Technology Pty Ltd

^c^Estimation based on co-localising markers published elsewhere

^d^Blastn against the *Triticum aestivum* TGACv1 genome sequence hits 4BL as of 5th Oct 2017

The 5BS QTL was selected for haplotype analysis, because it co-located with the genomic location for *Snn3*. Haplotypes for *Snn3* sensitivity gene to SnTox3 were constructed based on markers which satisfied three criteria: (1) they were located in close proximity to the QTL detected by these traits; (2) displayed pairwise linkage disequilibrium (LD) values ≥ 0.8 (Riaz et al. 2017b); and (3) were significantly associated with the SnTox3 sensitivity response. LD between all pairs of mapped markers was calculated using PopGen package in R (R_Core_Team 2013). The percentage of lines belonging to four groups based on the SnTox3 sensitivity scores (≥ 0, ≥ 1, ≥ 2, and ≥ 3) were plotted for each haplotype identified.

### Comparison of means

Statistical analyses were performed using JMP 10.0.0 (SAS Institute, CA, USA) or *R* (R_Core_Team 2013). All *t* test statistical analyses were deemed significantly different if the *P* value was < 0.01. For genotype group comparison analysis, accessions with the same number of resistance-associated alleles identified for each trait were grouped together. The disease severity resulting from SN15 and *toxa13* inoculations among the groups were compared. Significant differences between the groups were declared when the *P* < 0.01 with a ‘false discovery rate’ (FDR) test using pairwise.t.test(); weighted means of these groups were calculated by lm() function in R (R_Core_Team 2013).

## Results

### Removal of *SnToxA*, *SnTox1,* and *SnTox3* in *P. nodorum* SN15

**Fig. 1:**
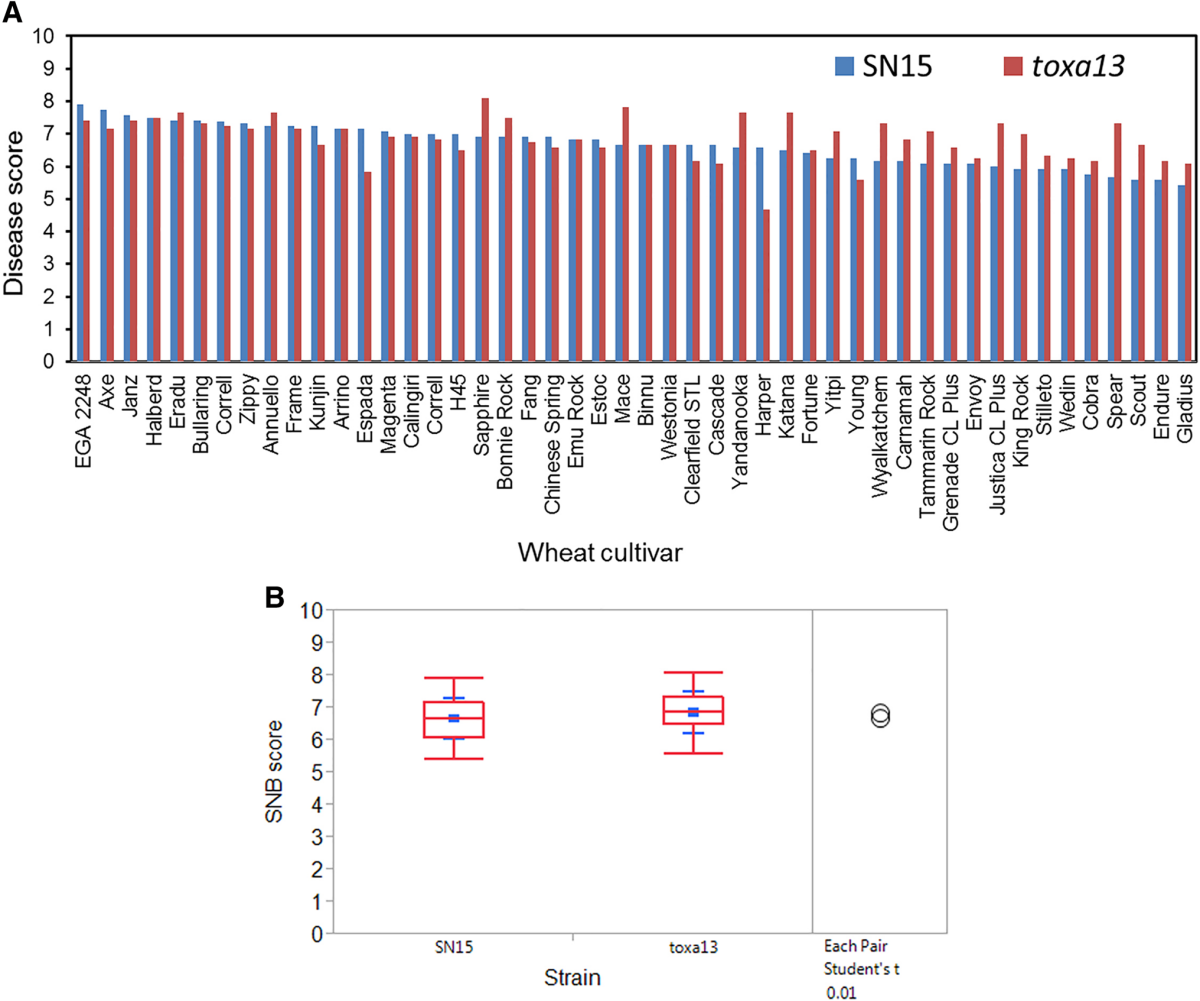
Pathogenicity assay of SN15 and *toxa13* on Australian wheat cultivars and Chinese Spring. **a** Disease distribution of SN15 and *toxa13*. **b** Pairwise comparison of average disease scores of SN15 and *toxa13* using a *t* test (left panel). Non-overlapping circles indicate that means are significantly different (*P* < 0.01). The red box plot is an indicator of median distribution, whereas blue annotation indicates the mean and standard deviation (right panel). The experiment was performed in biological triplicates (Supplemental data 1)

It was previously demonstrated that *P. nodorum toxa13* remained fully pathogenic on three major Australian wheat cultivars (Tan et al. 2015). In this study, we assayed an additional 47 Australian wheat cultivars and Chinese Spring with *toxa13* and SN15 (Fig. 1). For SN15, the average disease score of individual cultivars ranged from 5.41 to 7.92. For *toxa13*, the average disease score of individual cultivars ranged from 4.67 to 8.08 (Fig. 1a). The virulence of both strains was similar across all 48 lines (Fig. 1b). Given that numerous mapping studies had confirmed the role of *Tsn1*, *Snn1,* and *Snn3* (Friesen et al. 2008b; Liu et al. 2004a, 2006), it was surprising to observe that the removal of their interactions with SnToxA, SnTox1, and SnTox3 did not substantially reduce overall *toxa13* virulence. This indicates compensation through novel (but potent) effector–host dominant susceptibility gene interactions or the lack of a resistance mechanism contributed to SNB in the absence of SnToxA–*Tsn1*, SnTox1–*Snn1,* and SnTox3–*Snn3* interactions.

### The Vavilov wheat accessions provide a good source of seedling resistance

**Fig. 2:**
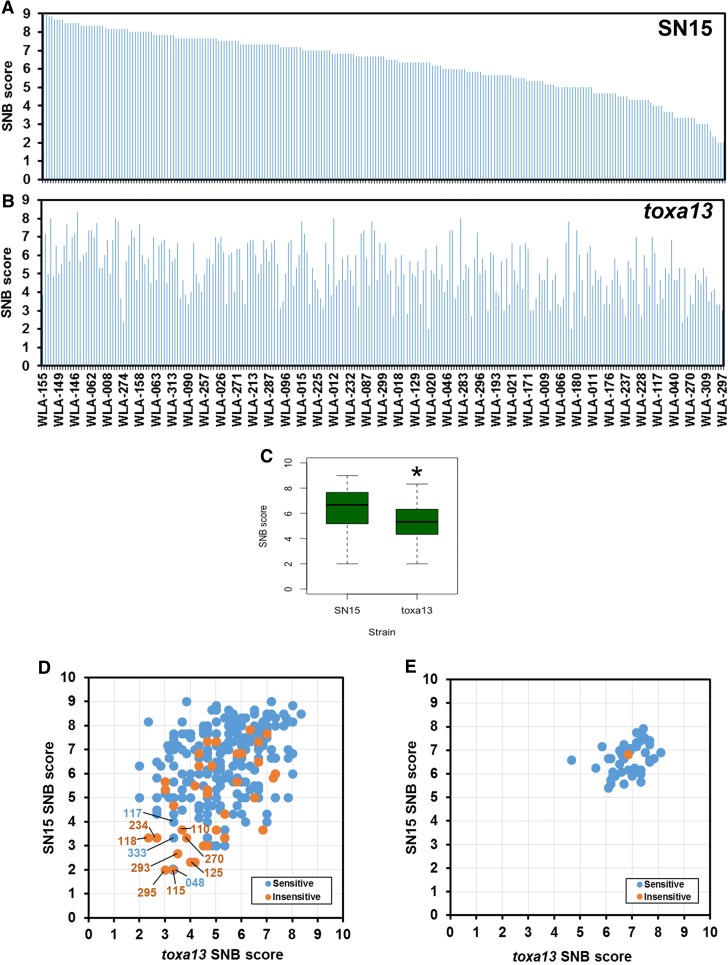
Comparison of SN15 and *toxa13* infection scores on the Vavilov wheat collection. Wheat accessions arranged from the highest to lowest SN15 SNB scores for **a** SN15 and **b** *toxa13*. **c** Average SN15 and *toxa13* disease scores. Asterisk denotes significant difference (*P* < 0.01) based on a weighted means *t* test. Dot plot comparisons of SN15 and *toxa13* SNB scores on **d** Vavilov accessions and **e** Australian wheat cultivars and Chinese Spring. SnToxA-, SnTox1- and SnTox3-insensitive accessions are shown. Selected UQ SSD line no (prefix ‘WLA’) is shown in panels **a**, **b,** and **d**. Accessions were considered insensitive with an average effector-sensitivity score of ≤ 1 (Tan et al. 2014)

The Vavilov wheat collection was then infected with SN15 and *toxa13* at the seedling stage to determine the SNB response with and without *SnToxA*, *SnTox1,* and *SnTox3*. For SN15, the average disease score of individual accessions ranged from 2.00 to 9.00 (Fig. 2a). For *toxa13*, the average disease score of individual accessions ranged from 2.00 to 8.33 (Fig. 2b). A comparison of the average disease score obtained using SN15 and *toxa13* revealed that SN15 (average disease score 6.29) is significantly more pathogenic than *toxa13* (average disease score 5.26) (*P* = 1.5e^−13^) (Fig. 2c). Scatterplot analysis highlighted that the Vavilov wheat collection (Fig. 2d) possesses a much broader range of resistance/susceptibility responses to both SN15 and *toxa13* compared with the Australian wheat cultivar collection (Fig. 2e). Both datasets possessed weak, but similar positive correlations between SN15 and *toxa13* disease scores for the Vavilov (*R* = 0.381) and Australian cultivars (*R* = 0.371). However, the absolute mean difference between SN15 and *toxa13* in the Vavilov panel (1.065) is significantly greater than in the Australian cultivars (0.558) (*P* = 5.4e^−10^). Analysis of the Vavilov scatterplot revealed striking evidence that SnToxA-, SnTox1-, and SnTox3-insensitive accessions displayed a lower susceptibility to SNB than those that possessed sensitivity to one or more effectors (Fig. 2d). Overall, the Vavilov wheat collection contained a higher degree of resistance to SNB. From this, we were able to identify 11 accessions with disease scores of ≤ 4 for SN15 and *toxa13*. This includes WLA-048, 110, 115, 117, 118, 125, 234, 270, 293, 297, and 310 (Fig. 2d; Supplemental data 2). Of these, eight accessions were insensitive to all three effectors (i.e., WLA-110, 115, 118, 125, 234, 270, 293, and 297) and three were sensitive to one or more effectors (i.e., WLA-048, 117 and 310). This highlights that response to SNB in the Vavilov wheat collection was much more variable than the Australian wheat cultivar collection.

### Contribution of effector sensitivity to SNB in the Vavilov wheat collection

**Fig. 3:**
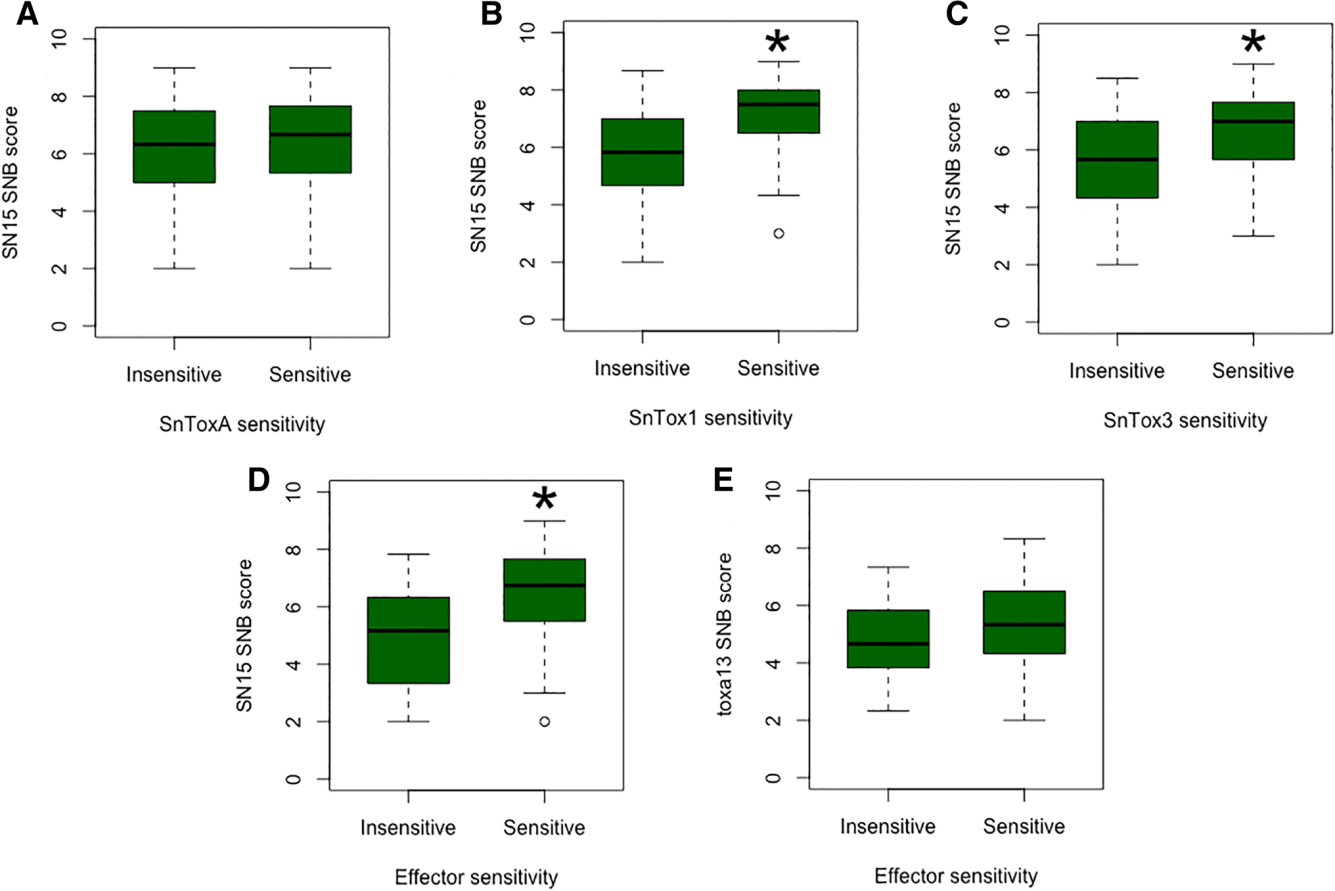
Contribution of effector sensitivity to SNB in the Vavilov wheat collection. **a** Average SN15 disease scores of SnToxA-sensitive versus insensitive accessions. **b** Average SN15 disease scores of SnTox1 sensitive versus insensitive accessions. **c** Average SN15 disease scores of SnTox3 sensitive versus insensitive accessions. **d** Average SN15 disease scores of SnToxA, SnTox1 and SnTox3 sensitive versus insensitive accessions. **e** Average *toxa13* disease scores of SnToxA, SnTox1 and SnTox3 sensitive versus insensitive accessions. Comparisons between groups were carried out using a weighted means *t* test with lm() function in *R*. Asterisk denotes significant difference (*P* < 0.01)

We then determined whether there were any correlations between effector sensitivity and susceptibility to SNB in the Vavilov wheat collection using the 253 accessions for which we had both infection and effector-sensitivity data. For SnToxA, 128 accessions were sensitive and 125 were insensitive to the effector. Average SN15 disease scores for SnToxA-sensitive and insensitive accessions were not significantly different (*P* = 0.116) based on a *P* < 0.01 significance cutoff (Fig. 3a). SnTox1-sensitive accessions (average disease score 7.12) were significantly more susceptible to SN15 than insensitive accessions (average disease score 6.44) (*P* = 5.4e^−13^) (Fig. 3b). Similar to SnTox1, SnTox3-insensitive accessions (*n* = 91) were significantly less susceptible to SN15 than accessions possessing the sensitivity phenotype (*n* = 162) (*P* = 1.4e^−8^) (Fig. 3c). Wheat accessions that were insensitive to SnToxA, 1 and 3 (*n* = 37) were significantly less susceptible to SN15 (average disease score 4.91) compared to accessions that possessed one or more effector susceptibility phenotypes (*n* = 216; average disease score 6.52) (*P* = 6.9e^−09^) (Fig. 3d). However, when all three effector genes were deleted (*toxa13*), no significant difference in disease rating was observed (*P* = 0.046) between sensitive (*n* = 216, average disease score 5.33) and insensitive accessions (*n* = 37, average disease score 4.83) (Fig. 3e). This suggests that effector interactions, at least for SnTox1–*Snn1* and SnTox3–*Snn3*, are major SNB determinants in the Vavilov wheat collection.

### QTL associated with SNB response using SN15 and *toxa13* isolates

We then investigated whether QTL corresponding to *Tsn1*, *Snn1,* and *Snn3* can be detected in the Vavilov wheat collection using phenotypic responses to SnToxA, SnTox1, and SnTox3 infiltrations. We infiltrated 259 accessions with SnToxA, SnTox1, and SnTox3 and scored the resulting chlorosis/necrosis symptoms 4-day post infiltration (Supplemental data 2). GWAS using the DArT/SNP marker data and effector infiltration responses detected QTL at the expected genomic locations. For SnToxA sensitivity, GWAS mapping revealed a significant association with the DArT marker 1168841 on the long arm of chromosome 5B (Table 1). The Vavilov wheat collection also displayed accession-specific sensitivity to SnTox1. Analysis of the phenotype via GWAS detected three significant marker–trait associations (1268227, 1195188, and 1099649) on chromosome 1BS at 7.025 cM (Table 1). Five markers on the short arm of chromosome 5B (position 4.119–6.153) displayed strong association with the SnTox3 sensitivity response (Table 1). These three QTL corresponded to the genetic location of *Tsn1*, *Snn1,* and *Snn3* for SnToxA, SnTox1, and SnTox3 sensitivity responses, respectively.

### GWAS of SN15 and *toxa13* SNB

We then performed GWAS using the SN15 and *toxa13* phenotypes to identify QTL associated with SNB in the Vavilov wheat collection. A total of 12 DArT markers were significantly associated with a disease response using SN15. These markers represented eight distinct QTL (Table 1). QTL detected on 1BS and 5BS corresponded with the genomic locations of *Snn1* and *Snn3*. *Tsn1* on 5BL was not detected, and this was somewhat expected since SnToxA sensitivity did not play a major role in seedling susceptibility to SN15 (Fig. 3a). An additional four QTL were detected using SN15 and were positioned on 4BS, 4AL, 3AL, and 7AS. However, blast analysis of the DArT marker 1676515 for the 4AL QTL revealed that the marker most strongly aligned to the long arm of chromosome 4B based on the TGACv1 Chinese Spring assembly. The 3AL QTL has the strongest effect on SN15 SNB (Table 1). For *toxa13*, a total of 3 DArT markers were significantly associated with a disease response. These markers represented three distinct QTL (Table 1). We were unable to detect QTL corresponding to the genomic location of *Snn1* and *Snn3*, 1BS, and 5BS, respectively (Table 1). Only three QTL were detected and these were on chromosomes 5AS, 2DL, and 7DL. The 5AS QTL has the strongest effect on *toxa13* SNB. It also corresponded to a QTL detected using SN15, whereas QTL on 2DL and 7DL were not detected using SN15. These results suggest that SnToxA, SnTox1, and SnTox3 (or their interactions with their matching dominant susceptibility genes) may interact epistatically with the loci on chromosomes 2DL and 7DL.

### Haplotype analysis of the 5BS QTL

**Fig. 4:**
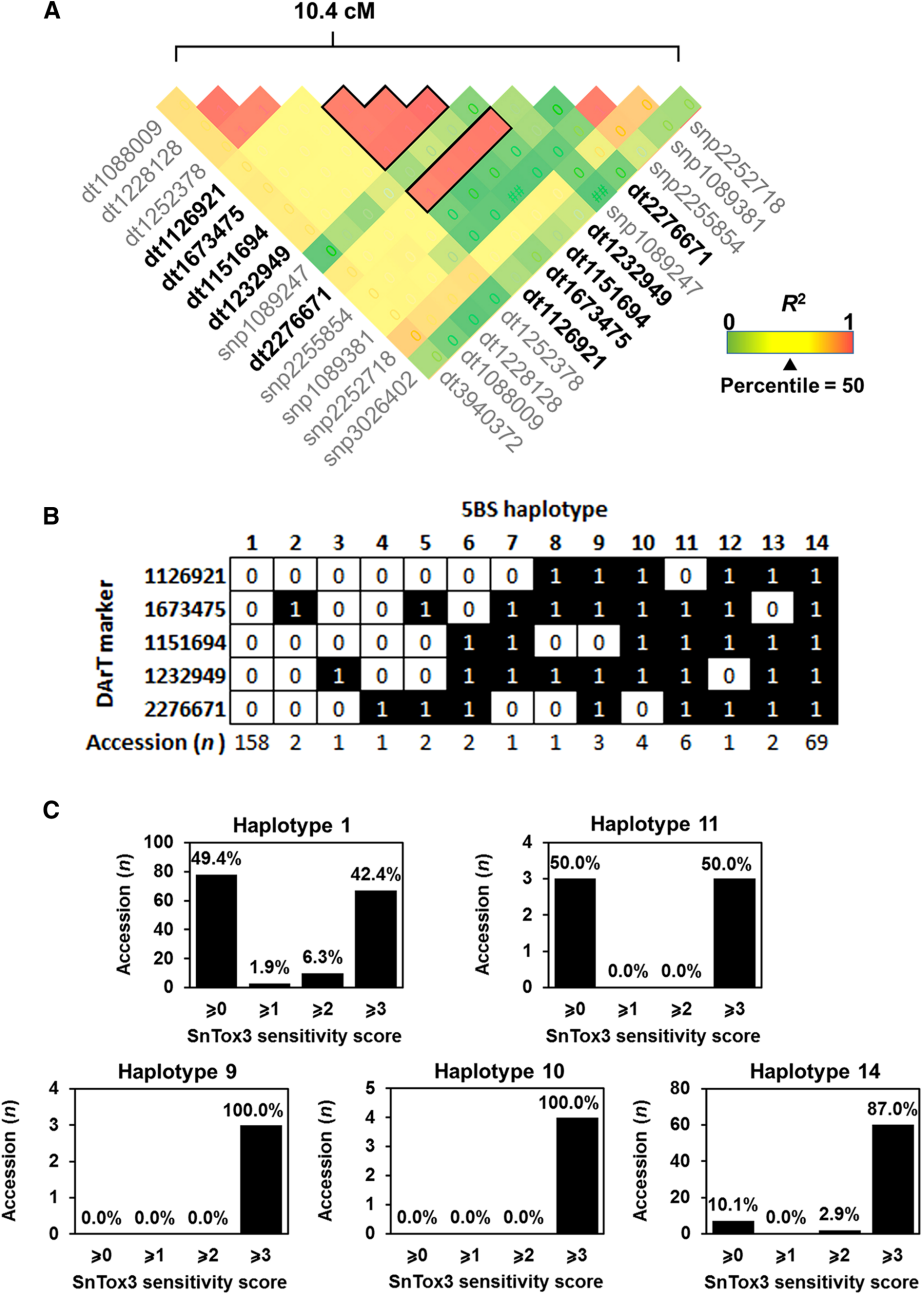
Haplotype analysis of the 5BS QTL associated with sensitivity to SnTox3. **a** Heat map showing pairwise linkage disequilibrium between marker pairs. Linkage disequilibrium blocks for five markers that were associated with the SnTox3 response are indicated. Prefixes; ‘dt’ indicates DArT markers and ‘snp’ for SNP markers. **b** 14 haplotypes were identified in the Vavilov wheat collection based on five linked markers. ‘1’ and ‘0’ designate two alleles for each marker. **c** Frequency of SnTox3 sensitivity in accessions across five major haplotypes

The *Snn3* locus confers sensitivity to SnTox3, yet the casual gene underpinning *Snn3* remains unknown. Since the 5BS QTL corresponded to the known genomic location of *Snn3*, we decided to perform a haplotype analysis on 5BS QTL markers that possessed significant association with SnTox3 sensitivity, in high LD (≥ 0.8) and located in close proximity to each other (Fig. 4a). As a result, five suitable DArT markers (1126921, 1673475, 1151694, 1232949, and 2276671) were selected for haplotype analysis. We were able to identify 14 haplotypes across 253 Vavilov wheat accessions based on the selected markers (Fig. 4b). Haplotypes 1 (*n* = 158) and 14 (*n* = 69) were the most frequent variants. We then selected five haplotypes that were present in three or more accessions and examined the frequency of SnTox3 sensitivity. It was observed that 48.7 and 50.0% accessions that possessed haplotypes 1 and 11, respectively, were considered insensitive to SnTox3 (sensitivity score ≤ 2). However, nearly, all accessions that possessed haplotypes 9, 10, and 14 were highly sensitive to SnTox3 (Fig. 4c). Thus, haplotypes 9, 10, and 14 are reliable marker combinations that can be used to screen for *Snn3*.

### Genotype group comparisons revealed quantitative resistance and susceptibility traits

**Fig. 5:**
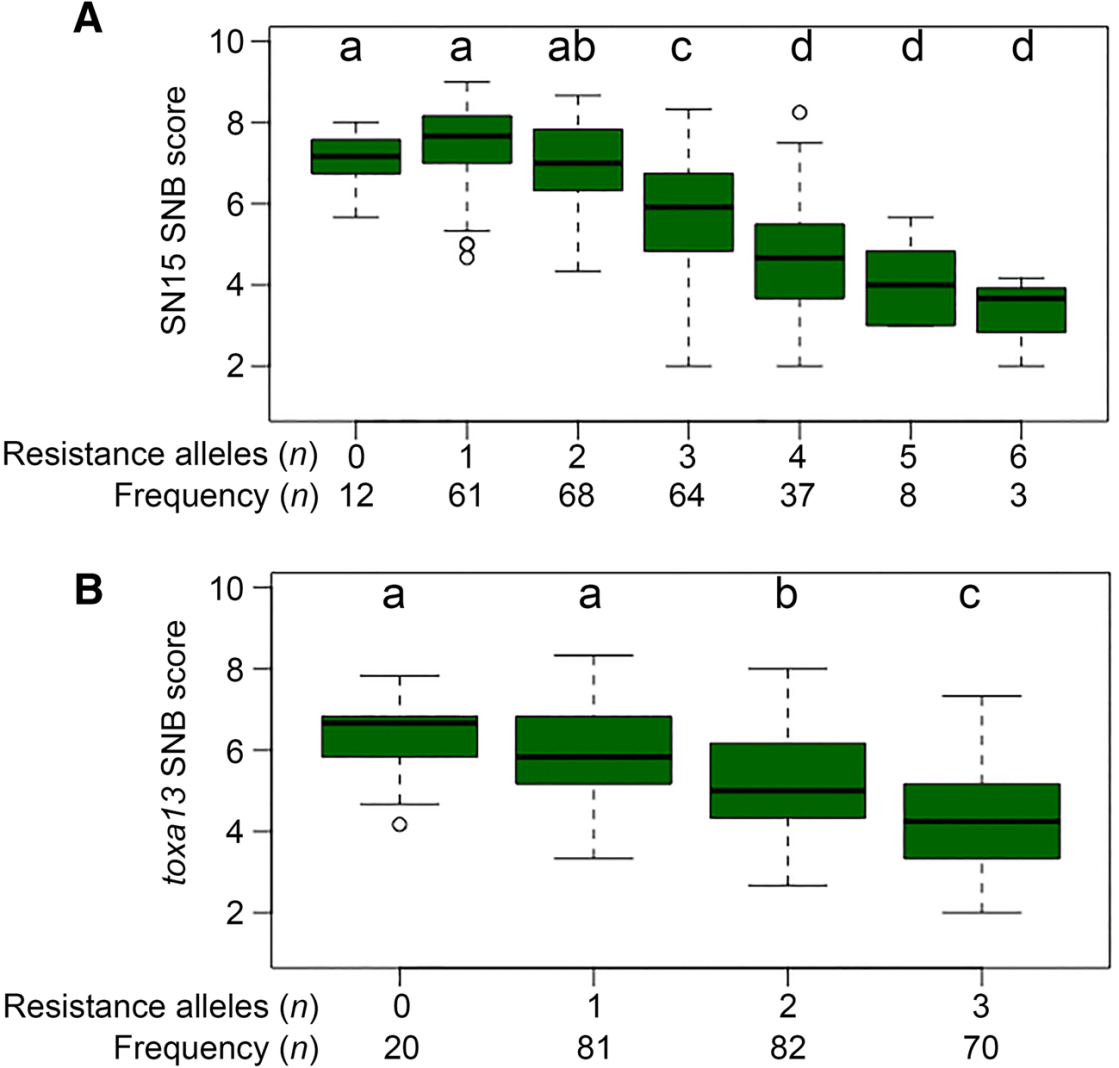
Effect of resistance-associated alleles stacking on **a** SN15 and **b** *toxa13* SNB. Comparisons between groups were carried out using a weighted means pairwise *t* test with an FDR test. Levels not connected by the same letter are significantly different (*P* < 0.01)

SNB is a complex disease largely controlled by the expression of multiple QTL conferring resistance and/or susceptibility to the fungus. However, the relationship between such QTL has not been thoroughly examined using diverse wheat accessions and *P. nodorum* strains carrying different effector profiles. We then tested the effect of an accumulation of alleles that are associated with resistance to SN15 and *toxa13* by assigning accessions from the Vavilov wheat collection to groups based on the number of resistance-associated alleles carried in each accession. Although eight QTL were detected in the Vavilov panel based on SN15 infection, the maximum number of resistance-associated alleles found in any one accession was six. Accessions that possessed three or more resistance-associated alleles were significantly more resistant to SN15 (Fig. 5a). However, accessions that carried four-to-six-resistance-associated alleles were similarly resistant to SN15 (*P* = 0.05–0.37). We then examined the contribution of the absolute number of resistance-associated alleles to *toxa13* infection. Accessions that accumulated two or more resistance-associated alleles displayed lower disease scores than accessions with only one and zero resistance-associated alleles (*P* = 4.2e^−4^–2.2e^−15^) (Fig. 5b).

### Spatial–temporal distribution of effector sensitivity in the Vavilov wheat collection

**Fig. 6:**
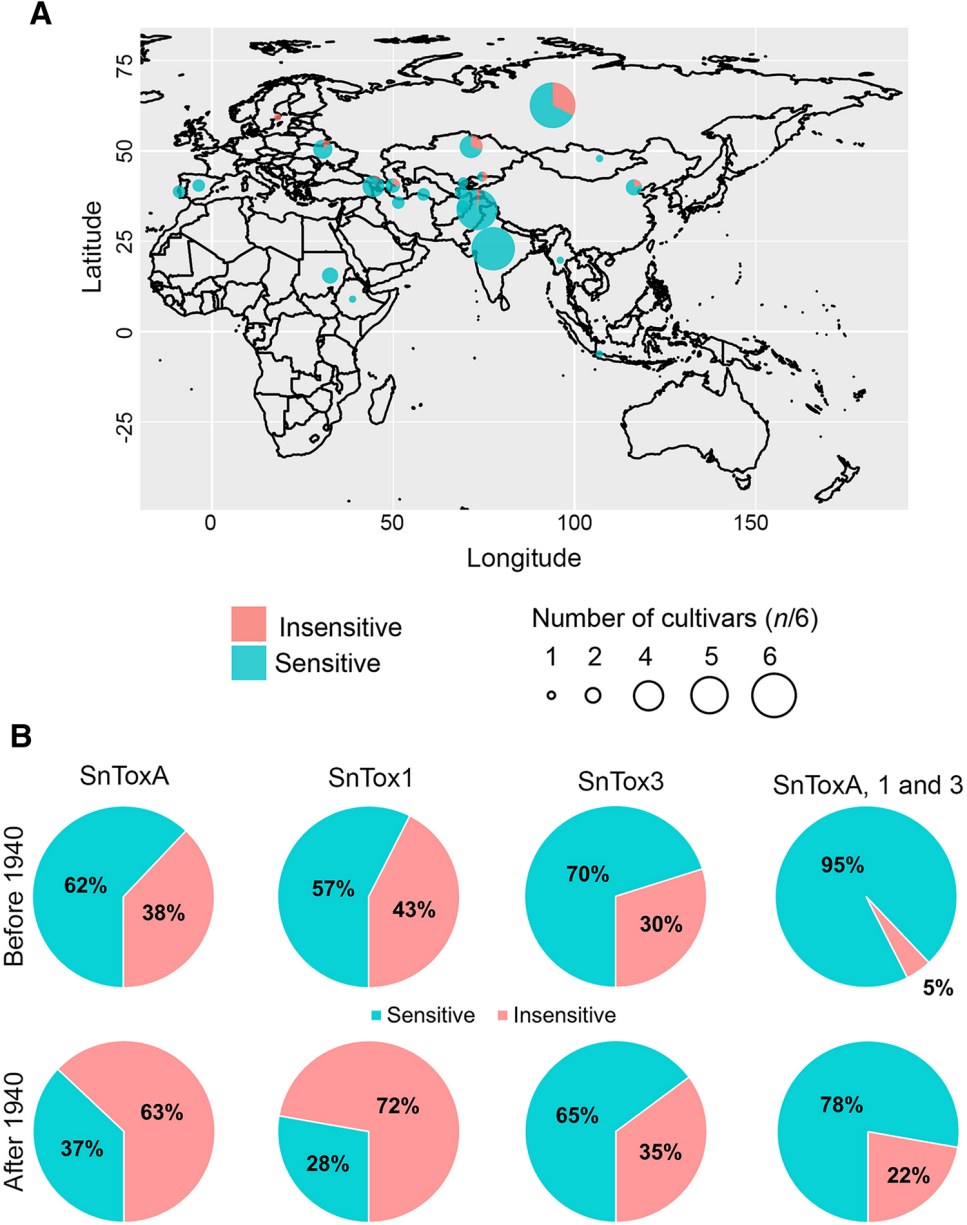
Spatial–temporal distribution of effector sensitivity in the Vavilov wheat collection. **a** World-wide distribution of wheat accessions that are insensitive to all three effectors. The distribution of SnToxA-, SnTox1- and SnTox3-only insensitive accessions are described in Supplemental data 4. **b** Distribution of effector sensitivity in Vavilov wheat accessions pre- and post-1940. The wheat accession WLA-082, registered in 1940, was included in the pre-1940 category. Accessions were considered insensitive with an average effector-sensitivity score of ≤ 1 (Tan et al. 2014)

We then investigated the geographical distribution of effector sensitivity/insensitivity in the Vavilov wheat collection. A total of 186 accessions possessed both spatial data and effector-sensitivity information. Russia (41), Kazakhstan (11), India (36), and Pakistan (32) accounted for over 64% of the 186 accessions. The geographical distribution of SnToxA-, SnTox1-, and SnTox3-sensitive wheat accessions were examined. We observed that a higher proportion of wheat accessions from Central Asian countries consisting of Russia (SnToxA, 70.7%; SnTox1, 73.2%; SnTox3, 48.8%) and Kazakhstan (SnToxA, 72.7%; SnTox1, 71.4%; SnTox3, 36.4%) were overall more insensitive to either SnToxA, SnTox1 or SnTox3 than accessions collected from South Asian countries such as India (SnToxA, 33.3%; SnTox1, 41.7%; SnTox3, 22.2%) and Pakistan (SnToxA, 40.6%; SnTox1, 28.1%; SnTox3, 31.2%) (Supplemental data 4). When insensitivity to all three effectors was taken into account, we observed that a high proportion of accessions registered from Russia (*n* = 13, 31.7%) and Kazakhstan (*n* = 3, 27.2%) were insensitive to all three effectors (Fig. 6a). In contrast, only one of 68 accessions from South Asia was insensitive to all three effectors. Effector insensitive lines were also collected from Armenia (*n* = 1 of 9), Azerbaijan (*n* = 1 of 4), China (*n* = 1 of 5), Kyrgyzstan (*n* = 1 of 2), Sweden (*n* = 1 of 1), and USA (*n* = 2 of 3).

We then examined the temporal distribution of effector sensitivity in the Vavilov wheat collection using 141 accessions with passport data that included year of registration at VIR (Fig. 6b). We divided these accessions into pre-1940 (*n* = 87) and post-1940 (*n* = 54). For SnToxA sensitivity, 38% of pre-1940 accessions were insensitive to the effector. After 1940, the proportion of SnToxA-insensitive accession increased to 63%. An increase in insensitivity to SnTox1 was similarly observed in post-1940 accessions. However, the proportion of registered wheat accessions that were sensitive to SnTox3 pre- and post-1940 remained similar. We then examined the temporal distribution of SnToxA-, SnTox1-, and SnTox3-insensitive accessions. About 5% of all wheat accessions registered pre-1940 were insensitive to all three effectors, whereas the proportion increased significantly to 22% in post-1940 accessions. This suggests that selection for effector insensitivity, particularly to SnToxA and SnTox1, may have occurred during the period when modern plant breeding programs were initiated.

## Discussion

SNB is a recalcitrant disease in many parts of the world. Progress in breeding for resistance has been modest (Vleeshouwers and Oliver 2014). The discovery of NEs improved our understanding of the complex interaction, but despite transfer of this knowledge to breeders, the best locally available cultivars in Australia only possess partial resistance to SNB (Shackley et al. 2014). This study was established with two goals. First, we sought a novel mapping resource to help dissect SNB, and second, we sought novel sources of resistance for breeders.

The Vavilov wheat collection is an excellent source of genetic diversity that was recently used to identify sources of resistance to tan spot and leaf rust of wheat (Dinglasan et al. 2018; Riaz et al. 2017a). In this study, we identified 11 accessions that displayed strong seedling resistance to SNB. It is not surprising that 8 of the 11 accessions are insensitive to all three effectors considering that effector insensitivity (to SnTox1 and SnTox3 at least) correlated with increased resistance to SNB. We have also identified accessions (e.g., WLA-268 and WLA-294) that are insensitive to all three effectors, but demonstrated high susceptibility to SNB. This suggests that novel effector–dominant susceptibility gene interactions are present in these accessions (Friesen et al. 2008a). It is conceivable that the fungus evolved functional redundancy of the NE system to compensate for the removal of dominant susceptibility genes in wheat through breeding. Efforts are underway to develop bi-parental crosses to elucidate the nature of SNB susceptibility in the absence of SnToxA–*Tsn1*, SnTox1–*Snn1,* and SnTox3–*Snn3* interactions.

We detected a total of ten unique SNB QTL by applying GWAS to the Vavilov wheat collection infected with SN15 and *toxa13*. Of these, seven were unique to SN15, two were unique to *toxa13* and one was common to both infections. For SN15, three QTL aligned with the genomic location of known effector-sensitivity genes—*Snn1*, *Snn2,* and *Snn3*. The SnTox3–*Snn3* interaction was also a major SNB determinant of infection using SN15. In addition to seedling assays, SnTox3–*Snn3* was also reported as an important determinant for SNB disease on adult wheat plants caused by natural infection (Ruud et al. 2017). However, several studies using modern bread wheat mapping populations indicate that SnTox3–*Snn3* is suppressed by SnTox1–*Snn1*, SnToxA–*Tsn1*, SnTox2–*Snn2*, SnTox5–*Snn5*, and SnTox6–*Snn6* interactions through epistasis (Friesen et al. 2008b, 2012; Gao et al. 2015; Phan et al. 2016). The mechanism of interplay between SnTox3–*Snn3* and other interactions is not known, but it can only be speculated that a combination of environmental and genetic factors play a role in epistasis. In this study, the SnTox3–*Snn3* interaction is expressed in the presence of SnTox1–*Snn1* and SnTox2–*Snn2* in SNB. It is unclear at this stage why epistasis between these interactions was not observed, but we suspect that the novel genetic makeup of the Vavilov wheat collection may be a contributing factor. SnTox1–*Snn1* interaction plays a key role in determining SNB on seedlings and adult plants (Liu et al. 2012; Phan et al. 2016). The importance of this interaction in SNB was validated via GWAS in the Vavilov panel through detection of a QTL corresponding with *Snn1*. To our surprise, we did not detect the SnToxA–*Tsn1* interaction in the Vavilov wheat collection infected with SN15. Gurung et al. (2014) detected a QTL on 5B using GWAS of a wheat panel infected with a *P. nodorum* strain that lacked SnTox3. They hypothesised that the 5B QTL corresponded to the genomic location of *Tsn1*. It is possible that the SnToxA–*Tsn1* may be masked by other interactions unique to SN15-infected Vavilov wheats. The QTL detected on 2DS corresponded to the genetic location of *Snn2* that confers sensitivity to SnTox2. SnTox2 is a small (7–10 kDa) proteinaceous effector partially characterised from the *P. nodorum* Sn6 isolate (Friesen et al. 2007). Genetic analysis indicates that SN15 also possesses *SnTox2* (Friesen et al. 2008b). It was expected that the 2DS QTL will compensate in SNB for the loss of SnToxA–*Tsn1*, SnTox1–*Snn1,* and SnTox3–*Snn3* interactions, but this was not the case with the Vavilov panel. However, the *Snn2*-associated 2DS QTL was also detected from *toxa13* infection in a double haploid mapping population derived from a cross between Calingiri and Wyalkatchem (Phan et al. 2016).

In addition to QTL on 1BS, 5BS, and 2DS that are associated with genes that confer sensitivity to known NEs, the QTL on 4AL is located on 4BL based on Blastn analysis of the DArT marker 1676515 against the wheat genome (Clavijo et al. 2017). The SnTox5 effector-sensitivity gene *Snn5* is located on 4BL (Friesen et al. 2012). Genetic analysis identified an SSR marker Xwmc349 that mapped 2.8 cM proximal to the *Snn5* locus (Friesen et al. 2012). In this study, it was observed that 1676515 mapped 6 cM proximal to Xwmc349 on the wheat genome. This indicates that that SnTox5–*Snn5* interaction is important in SN15 but not *toxa13* SNB. Other QTL observed on 3AL, 4BS, 5AS, and 7AS were not associated with any known effector-sensitivity/resistance-associated gene loci. A study by Aguilar et al. (2005) on glume blotch resistance on a recombinant inbred population produced from a cross of the winter wheat variety Forno and winter spelt variety Oberkulmer identified QTL on 3A, 4B, and 5A. A QTL on 4BS was also detected for flag leaf resistance using the EGA Blanco × Millewa double haploid population (Francki et al. 2011). A QTL on 7AS was also observed using a recombinant inbred line population derived from a cross between Salamouni and Katepwa when infected with the Swiss *P. nodorum* isolate Sn99CH 1A7a (Abeysekara et al. 2012).

We hypothesised that deletion of the three major effector genes in SN15 would reveal unique QTL that were masked by the large effects associated with *Tsn1*, *Snn1,* and *Snn3*. This strategy effectively uncovered QTL on 2DL and 7DL that were not detected using SN15. The QTL detected on 2DL was also reported by Gurung et al. (2014) in their GWAS study. The 2DL QTL is the probable genomic location of *Snn7*. *Snn7* is a dominant susceptibility gene to SnTox7, an effector partially characterised from the *P. nodorum* Sn6 isolate (Shi et al. 2015). Like SnTox2, SnTox7 has not been isolated and designated to a coding gene. However, biochemical characterisation revealed that the protein is heat stable and less than 30 kDa (Shi et al. 2015).

The importance of *Snn3* in SnTox3-mediated SNB is well-documented (Liu et al. 2009; Ruud et al. 2017). However, the identity of *Snn3* remains unknown. It is not surprising that efforts are underway to develop genetic markers for high-resolution mapping of *Snn3* (Shi et al. 2016). In this study, we have identified five markers that were significantly associated with SnTox3 sensitivity. Of these, four displayed significant association with SN15 SNB on the Vavilov wheat collection. When we performed haplotype analysis using the five linked markers, 14 distinct haplotypes were evident in the Vavilov wheat collection. Marker combinations of haplotypes 9, 10, and 14 can accurately diagnose SnTox3 sensitivity, at least in Vavilov wheats. Thus, haplotype analysis of the 5BS QTL provides useful information towards cloning of *Snn3* and marker-assisted selection in conjunction with SnTox3 bioassays to improve SNB resistance.

Results from genotype group comparisons partially explained the additive and complex nature of SNB (Friesen and Faris 2010; Nelson and Gates 1982). Here, we demonstrate that an accumulation of resistance-associated alleles in wheat accessions additively confer resistance to SNB in addition to SnTox1 and SnTox3 insensitivity. Therefore, we can conclude that a combination of effector insensitivity and stacking of resistance-associated alleles in cultivars presents the best option for optimal SNB resistance. For breeding purposes, pyramiding up to four independent resistance-associated alleles in conjunction with effector insensitivity will raise the resistance levels in wheat. Information from this study, once validated with adult plant infection, will assist cereal breeders to formulate optimal QTL combinations in their breeding lines through marker-assisted selection to minimise the impact of SNB.

The availability of passport data for accessions in the Vavilov wheat collection revealed intriguing information on the geographical distribution of effector sensitivity. We observed that accessions from the former Soviet Union overall displayed a high proportion of insensitivity to SnToxA, SnTox1, and SnTox3, as opposed to accessions from South Asia. The climatic conditions differ greatly between these two regions. Halama and Lacoste (1992) demonstrated that the production of sexual fruiting bodies known as pseudothecia in *P. nodorum* are maximal at 10 °C and absent at temperatures above 14 °C. From this, Vergnes et al. (2006) proposed that the production of pseudothecia is promoted during the snow melting period. We can only speculate that this event may be conducive in triggering maximal production of airborne ascospores that are responsible for infection of young wheat leaves grown in colder regions of Central Asia. Taken collectively, climatic requirements in Central Asia are more conducive for *P. nodorum* dissemination than South Asia. This may have led to inadvertent selection for wheat lines that possess the genetic potential for improved SNB resistance. Analysis of the *P. nodorum* population from Central Asia revealed that *SnTox1* was highly prevalent (89%) in all isolates (McDonald et al. 2013). Over 70% Vavilov wheat accessions from Russia and Kazakhstan were insensitive to SnTox1, thus suggesting regional adaptation for selection against a prevalent effector. However, knowledge on the distribution of *P. nodorum* effectors in South Asia is lacking (McDonald et al. 2013).

Temporal analysis suggests evidence of inadvertent selection for NE insensitivity particularly to SnToxA and SnTox1, but not SnTox3 (Oliver and Solomon 2008). It is interesting to note that SnTox3 sensitivity is highly prevalent in Australian cultivars (Tan et al. 2014) and CIMMYT and ICARDA breeding material (Supplemental data 5). It is possible that *Snn3* may be closely linked to other desirable breeding traits or its direct removal incurs a trait penalty other than yield (Oliver et al. 2014). The importance of SnTox1–*Snn1* in seedling and adult plant SNB has been thoroughly demonstrated in several studies (Liu et al. 2004b, 2012; Phan et al. 2016). Thus, SnTox1 insensitivity is a likely target for selection in breeding programs post-1940. Like SnTox1, the reduction in the proportion of SnToxA-sensitive accessions registered post-1940 is presumably due to selective breeding that results in indirect selection against *Tsn1*. However, SnToxA sensitivity in the Vavilov panel does not play a major contributing role in seedling SNB in this study. There are two possible explanations. First, SnToxA has been previously demonstrated as a key virulent determinant of *P. nodorum* on *Tsn1* wheat lines (Friesen et al. 2006). Furthermore, the SnToxA–*Tsn1* interaction was expressed in adult plants during field infection (Friesen et al. 2009). It is possible that specific environmental and developmental conditions affect the expression of the SnToxA–*Tsn1* interaction. Studies are currently underway to examine the importance of field-based adult plant infection of the Vavilov wheat collection with *P. nodorum*.

Finally, the tan spot (syn. yellow spot) of wheat fungus *Pyrenophora tritici*-*repentis* (*Ptr*) also uses effectors to cause chlorosis and necrosis on wheat lines that carry dominant susceptibility genes. Analysis of the *Ptr* genome identified a copy of *ToxA* that is near-identical to *P. nodorum* (Friesen et al. 2006). It was hypothesised that *Ptr* may have acquired ToxA through lateral gene transfer, presumably from *P. nodorum*. This event may have occurred just before 1941, therefore, rendering *Ptr* pathogenic on *Tsn1* wheats (Friesen et al. 2006). Tan spot has become increasingly damaging on wheat in the 1960s and the 70s (Oliver and Solomon 2008). For example, an explosion in the population of *Ptr* carrying *ToxA* was observed in the 70s coinciding with the mass plantings of *Tsn1* wheat in Canada (Lamari et al. 2005). Recently, Dinglasan et al. (2018) observed a positive correlation between the severity of tan spot at the seedling stage and ToxA sensitivity in the Vavilov panel. Thus, we hypothesised that selection for effector insensitive wheat lines post-1940 may have been performed on more than one front. In addition, *ToxA* has also been detected in some isolates of *Parastagonospora avenae* (causes SNB), but has yet to be characterised for its role in virulence on *Tsn1* wheats (McDonald et al. 2013). More recently, *ToxA* was identified in *Bipolaris sorokiniana,* the causal agent of spot blotch of wheat (McDonald et al. 2018). *B. sorokiniana* containing the orthologous gene is more virulent on *Tsn1* wheat than a *ToxA*-negative isolate (McDonald et al. 2018). Unlike *P. nodorum*, *B. sorokiniana* prefers a warm humid climate for optimal infection and is considered a major pathogen of wheat in South Asia (Gupta et al. 2017). It is not known at this stage if *ToxA* is prevalent in Indian *B. sorokiniana* isolates; however, the high prevalence of SnToxA-sensitive Vavilov wheats from South Asia is a potential driver of SnToxA–*Tsn1*-mediated virulence.

Whilst the Vavilov Institute houses over 38,000 wheat accessions, it is only feasible for us to examine 260 of these accessions for effector sensitivity and susceptibility/resistance to *P. nodorum* strains differing in effector gene profiles. From this, we were able to gain insight of spatial and temporal distribution of effector sensitivity from accessions from 28 different countries that were registered between 1922 and 1990. It is difficult to further correlate effector insensitivity between regions and year of registration due to missing data. However, access to the main collection at VIR will provide greater clarity on information on the possible origin of effector sensitivity. Despite the limited number of Vavilov wheats sampled in this study, we (1) identified evidence of sources of strong resistance to SNB and (2) determined the quantitative importance of effector sensitivity (to SnTox1 and SnTox3) in SNB through multiple effector gene deletions in *P. nodorum* and genetic diversity in wheat. The accessions of interest can be used to develop bi-parental mapping populations to further characterise the resistance alleles in parallel with backcrossing them into modern bread wheat to minimise the impact of SNB on wheat.

### **Author contribution statement**

KCT conceived and designed the experiments. KCT and HTTP wrote the manuscript. LH, ED, and RPO provided intellectual feedback. LH, ED, KR, and RPO edited the manuscript. HTTP, KR, SB, and EF performed all experiments. Results were analysed by KCT and HTTP.

## Electronic supplementary material

Below is the link to the electronic supplementary material.

Supplementary material 1 Supplemental data 1 SN15 and *toxa13* infection scores on Chinese Spring and 47 Australian wheat cultivars (XLSX 18 kb)

Supplementary material 2 Supplemental data 2 Effector sensitivity and SNB scores on the Vavilov wheat collection (XLSX 45 kb)

Supplementary material 3 Supplemental data 3 Manhattan plots displaying marker–trait associations from GWAS of the Vavilov collection for response to **a** SnToxA sensitivity, **b** SnTox1 sensitivity, **c** SnTox3 sensitivity, **d** SN15 SNB and **e** *toxa13* SNB (JPEG 1219 kb)

Supplementary material 4 Supplemental data 4 Spatial distribution of effector sensitivity in the Vavilov wheat panel. Proportion of accessions that lacked sensitivity to **a** SnToxA, **b** SnTox1, **c** SnTox3 and **d** all three effectors are indicated in pink. Accessions were considered insensitive with an average effector-sensitivity score of ≤ 1 (JPEG 3716 kb)

Supplementary material 5 Supplemental data 5 Effector sensitivity of modern wheat lines from CIMMYT to ICARDA (XLSX 35 kb)
