# Supplementary figures and images for: Novel sources of resistance to Septoria nodorum blotch in the Vavilov wheat collection identified by genome-wide association studies
Source: Theor Appl Genet. 2018 Feb 22;131(6):1223–38. doi: 10.1007/s00122-018-3073-y (PMC5945755; doi:10.1007/s00122-018-3073-y)

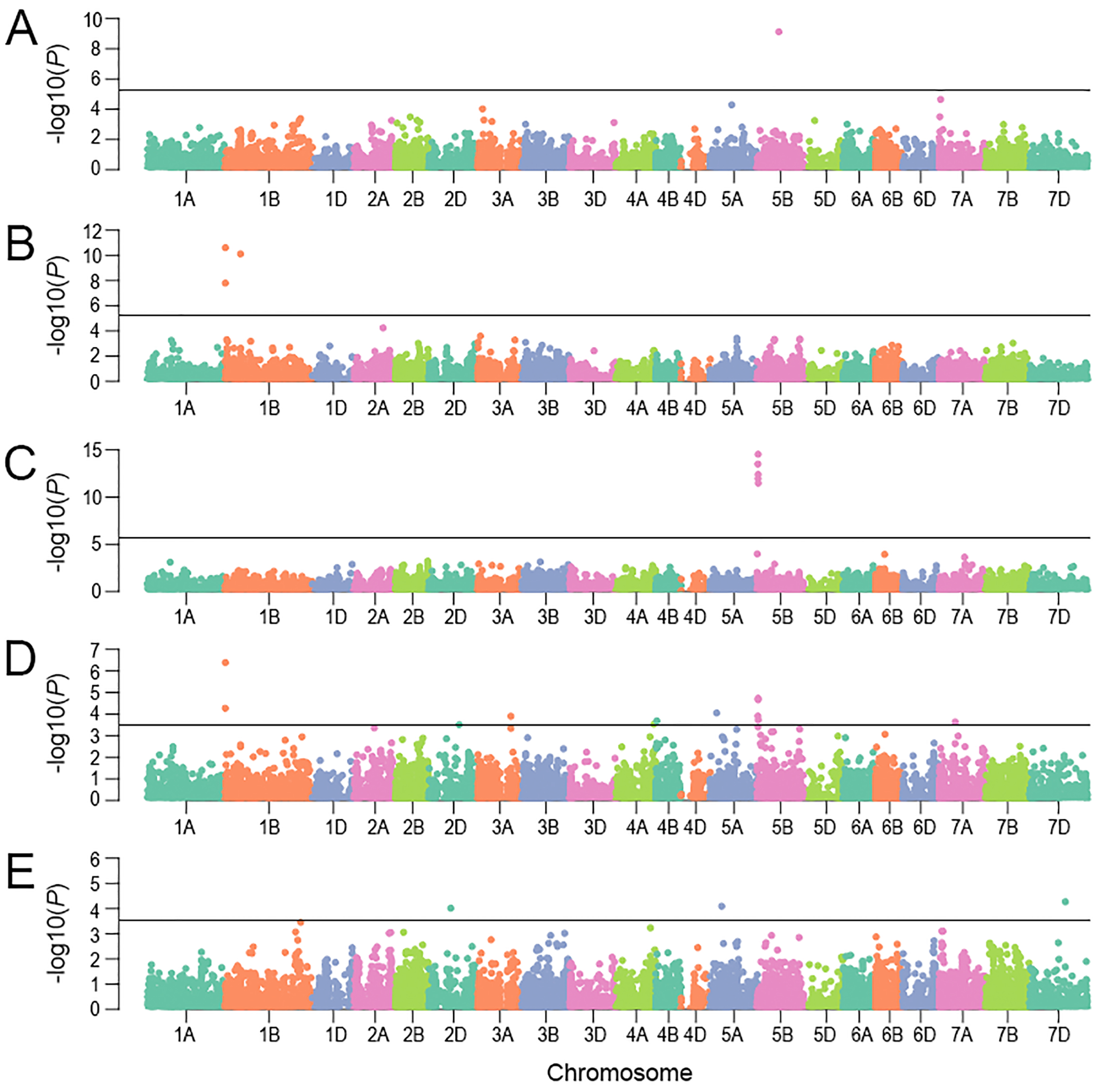

Supplement: Supplementary file 3 — Supplementary material 3 Supplemental data 3 Manhattan plots displaying marker–trait associations from GWAS of the Vavilov collection for response to a SnToxA sensitivity, b SnTox1 sensitivity, c SnTox3 sensitivity, d SN15 SNB and e toxa13 SNB (JPEG 1219 kb) [file 122_2018_3073_MOESM3_ESM.jpg]

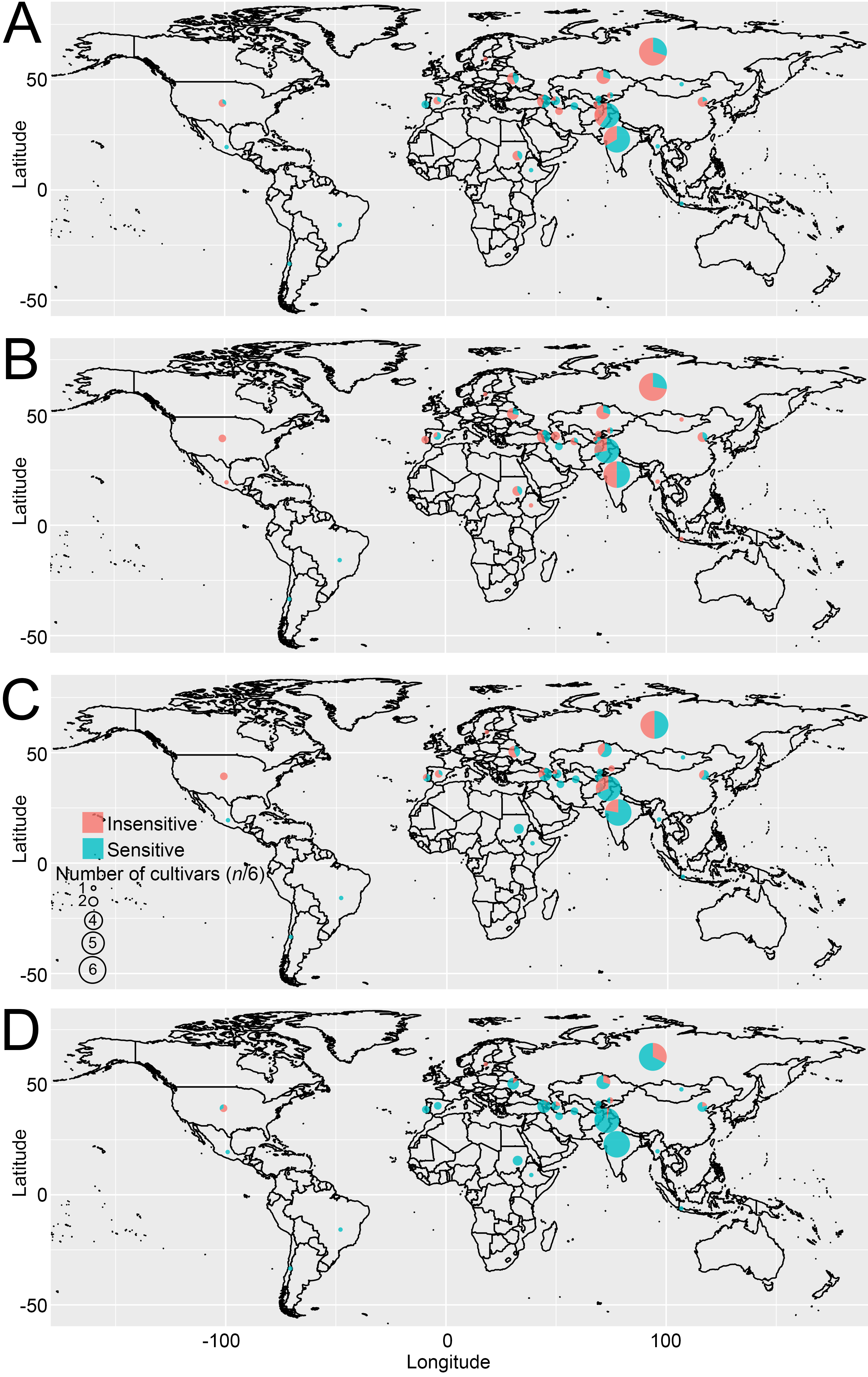

Supplement: Supplementary file 4 — Supplementary material 4 Supplemental data 4 Spatial distribution of effector sensitivity in the Vavilov wheat panel. Proportion of accessions that lacked sensitivity to a SnToxA, b SnTox1, c SnTox3 and d all three effectors are indicated in pink. Accessions were considered insensitive with an average effector-sensitivity score of ≤ 1 (JPEG 3716 kb) [file 122_2018_3073_MOESM4_ESM.jpg]
